# Noncoding RNAs: Possible Players in the Development of Fluorosis

**DOI:** 10.1155/2015/274852

**Published:** 2015-08-03

**Authors:** Atul P. Daiwile, Saravanadevi Sivanesan, Alberto Izzotti, Amit Bafana, Pravin K. Naoghare, Patrizio Arrigo, Hemant J. Purohit, Devendra Parmar, Krishnamurthi Kannan

**Affiliations:** ^1^Environmental Health Division, National Environmental Engineering Research Institute (NEERI), CSIR, Nagpur 440020, India; ^2^Department of Health Sciences, University of Genoa, No. 5, 16126 Genoa, Italy; ^3^IRCCS AOU San Martino IST, No. 10, 16132 Genoa, Italy; ^4^CNR Institute for Macromolecular Studies, No. 6, 16149 Genoa, Italy; ^5^Environmental Genomics Division, National Environmental Engineering Research Institute (NEERI), CSIR, Nagpur 440020, India; ^6^Developmental Toxicology, Indian Institute of Toxicology Research, Lucknow 226001, India

## Abstract

Fluorosis is caused by excess of fluoride intake over a long period of time. Aberrant change in the Runt-related transcription factor 2 (RUNX2) mediated signaling cascade is one of the decisive steps during the pathogenesis of fluorosis. Up to date, role of fluoride on the epigenetic alterations is not studied. In the present study, global expression profiling of short noncoding RNAs, in particular miRNAs and snoRNAs, was carried out in sodium fluoride (NaF) treated human osteosarcoma (HOS) cells to understand their possible role in the development of fluorosis. qPCR and in silico hybridization revealed that miR-124 and miR-155 can be directly involved in the transcriptional regulation of Runt-related transcription factor 2 (RUNX2) and receptor activator of nuclear factor *κ*-B ligand (RANKL) genes. Compared to control, C/D box analysis revealed marked elevation in the number of UG dinucleotides and D-box sequences in NaF exposed HOS cells. Herein, we report miR-124 and miR-155 as the new possible players involved in the development of fluorosis. We show that the alterations in UG dinucleotides and D-box sequences of snoRNAs could be due to NaF exposure.

## 1. Introduction

The global prevalence of fluorosis is a matter of great concern. Chronic exposure of high concentration of fluoride (>1.5 mg/L) may result in skeletal and dental deformities [1]. At present, 25 countries around the world have endemic to fluorosis [1]. In India, the health of approximately 62 million people is at risk due to high concentration of fluoride in drinking water/diet and so forth [1]. Preventive measures against fluorosis rely on the intake of low fluoride diet/water to reduce the ingestion of fluoride inside the body [1, 2]. However, these preventive measures were not successful in most of the underdeveloped and developing counties due to high cost associated with the defluoridation techniques [1, 2]. Available drug therapy against fluorosis includes supplementations with aluminum, magnesium, calcium, amino acid, and vitamins (C, D, and E) [1, 3]. These supplementations can stimulate calcium bone mineralization, activation of phagocytes/osteoclasts and antioxidant enzymes to combat oxidative stress induced by fluoride [2, 3]. However, the therapeutic effects incurred due to these supplements are unstable and reversible [2, 3].

No concurrent therapies are available against fluorosis due to limited information on the expression of aberrant posttranscriptional cascades involved in the development of skeletal and dental deformities [3]. Intake of high concentration of fluoride is reported to impede skeletal formation and remodeling processes due to failure in the signal transduction between the osteoblast and osteoclast cells through receptor activator of nuclear factor *κ*-B ligand (RANKL), receptor activator of nuclear factor *κ*-B (RANK), and osteoprotegerin (OPG) system [4]. RANKL/RANK signaling positively regulates osteoclast differentiation and promotes bone remodeling, whereas OPG/RANKL signaling protects the skeleton from the bone [4–7]. Aberrant variations in the RANKL/RANK/OPG system limits the availability of free calcium required for osteocalcin (bone gamma-carboxyglutamic acid-containing protein; BGLAP) mediated bone mineralization, leading to the excess of fluoride deposition inside the bones [6, 8].

The research on posttranscriptional gene-expression regulation raveled the pivotal role of short noncoding RNAs (miRNA, snoRNA, etc.) in the response of cell against environmental factors. In particular the miRNAs are the deeply investigated class of noncoding RNAs. But, limited reports entail that miRNAs can regulate bone formation and remodeling processes [9–11]. miR-204 and miR-211 were reported as the negative regulators of the gene involved in osteoblast activation, RUNX2 [9]. Similarly, the RUNX2-targeting miRNAs were reported to impede osteoblast differentiation [10, 11]. Thus, it is likely that miRNAs can be involved in the posttranscriptional regulation of other important signaling cascades (e.g., RANKL/RANK/OPG system and BGLAP) required for bone formation and remodeling processes. Less information is available on the effects of environmental factors on short noncoding RNAs, the small nucleolar RNAs (snoRNAs) [12]. Small nucleolar RNAs (snoRNAs) are a group of small RNA that principally regulate chemical alterations in other noncoding RNAs such as transfer RNAs (tRNAs), ribosomal RNAs (rRNAs), and small nuclear RNAs (snRNAs) [13]. snoRNAs are divided into two main classes depending upon the presence of C/D box and H/ACA box sequences [13]. C/D box snoRNAs are primarily involved in 2′-*O*-ribose methylation, whereas H/ACA box snoRNAs are involved in the pseudouridylation of noncoding RNAs [13].

Deleterious effects of fluoride on the expression levels of noncoding RNAs have not been studied. Herein, for the first time, we report a global expression profiling of noncoding RNAs (miRNAs and small nucleolar RNAs (snoRNAs)) in sodium fluoride (NaF) exposed human osteosarcoma (HOS) cells. Compared to control, analysis using Affymetrix miRNA 3.0 array platform (5639 human probes) revealed differential expressions of signature miRNAs and snoRNAs after sodium fluoride treatment (8 mg/L and 20 mg/L) to HOS cells. Further validation using real time PCR illustrated the upregulation of miR-124 and miR-155, whereas the expression levels of RANKL, BGLAP, and RUNX2 genes were decreased in NaF treated cells. OPG, a soluble decoy of RANKL, was upregulated in NaF treated cells. Data obtained from in silico hybridization analysis revealed that miR-124 and miR-155 could be directly involved in the posttranscriptional regulation of RUNX2 and RANKL genes. Compared to the terminal C/D box, microarray analysis revealed more marked effect of fluoride exposure on the expression levels of internal C/D boxes of snoRNAs. In addition, D-box, a pivotal element in methylation process, was found to be strongly influenced by NaF. Our results suggest the possible involvement of miR-124 and miR-155 in the development of fluorosis. In addition, high sensitivity of NaF to D-box of snoRNAs suggests the posttranscriptional regulation of hypo/hypermethylated and pseudourydilated rRNAs that could be involved in bone formation and remodeling processes.

## 2. Material and Methods

### 2.1. Cell Lines

Human osteosarcoma (HOS) cell line was purchased from National Centre for Cell Science (NCCS) Pune, India. Cells were resuspended in Minimum Essential Medium (MEM) supplemented with 10% fetal bovine serum, 100 U/mL penicillin, 50 *μ*g/mL streptomycin, and 25 *μ*g/mL Fungizone. Cells were maintained at 37°C in a humidified atmosphere with 5% CO_2_.

### 2.2. Cell Viability Assay

Cell viability was assessed using 3-(4,5-dimethylthiazol-2-yl)-2,5-diphenyltetrazolium bromide (MTT) assay. Cells (10,000 cells/well) were seeded in a 96-well plate for 24 h. After 24 h of seeding, HOS cells were exposed to different concentrations of NaF (10 mg/L to 250 mg/L) and further incubated for 24 h. After 24 h of exposure, MTT solution (5 mg/L in PBS) (Invitrogen, USA) was added and cells were incubated at 37°C for 3 h. Media were discarded and cells were resuspended in 200 *μ*L of DMSO (Sigma Aldrich, USA) and analyzed at 565 nm. Lethal concentration 50 (LC_50_) of NaF was calculated using the dose response curve analysis.

### 2.3. NaF Treatment

HOS cells were exposed to sublethal concentrations (8 mg/L (1/5th) and 20 mg/L (1/2nd) of 24 h LC_50_) of NaF (Sigma Aldrich, USA) for 30 days; subsequent controls were maintained. 50,000 cells/mL were seeded in cell flask, followed by the treatment of sodium fluoride. Cells were subcultured after 3 days and NaF treatment was given after every passage and culture media change. Finally, cells were harvested in Trizol reagent (Invitrogen, USA).

### 2.4. miRNA Microarray

Total RNA was isolated using Absolutely RNA Kit (Stratagene, USA), according to the manufacturer's recommendations. Quality of RNA was checked using Agilent Bioanalyzer 2100 (Agilent Technologies Inc., USA). Affymetrix miRNA 3.0 array platform was used to elucidate the variations in the expression profiles of 5639 human noncoding mature RNA probes after NaF treatment to HOS cells. Hybridization was carried out at 48°C for 16 h at 60 rpm. Probe intensities were measured using GeneChip Scanner 3000 7G (Affymetrix, USA). All the original microarray data (CEL files) for the experiments was preprocessed using RMA (Robust Multichip Average) algorithm that consists of three steps: a background adjustment, quantile normalization, and summarization. The raw data normalization has been performed by selecting “organism human” in Expression Console tool. All above procedures were done by selecting default RMA algorithm, data adjustment, and background correction (GABG) in Affymetrix Expression Console 1.2.1.20. Box whisker plot (see Supplementary data; S1 available online at http://dx.doi.org/10.1155/2015/274852) and quality control analyses were carried out using Expression Console software. Normalized intensity files were exported from Expression Consoles tool in txt format. In next step, all normalized experimental data were imported in GeneSpring GX 12.5 software for the differential miRNA expression, fold change analysis, and cluster analysis. Statistical analysis was performed for the identification of differentially expressed miRNA from two groups (Dose_1/5 (8 mg/L) versus control and Dose_1/2 (20 mg/L) versus control) (Supplementary Table; S2). One-way ANOVA method was applied for assessing the statistically significant differentially expressed miRNAs for two different treated experiment groups with reference to control. The *P* value cut-off 0.05 was considered statistically significant. miRNA microarray data has been submitted in NCBI GEO, accession number GSE57550.

### 2.5. In Silico Hybridization Analysis

mirTarBase and miRANDA software were used to identify functionally validated targets of miR-124 and miR-155. Furthermore, differentially expressed miRNAs were screened for their possible putative targets using in silico hybridization analysis. Possible binding sites of miR-124 and miR-155 with the RUNX2 and RANKL were identified.

### 2.6. Real Time qPCR

Screened miRNAs and genes were validated using quantitative qPCR. miRNAs were isolated using mirPremier microRNA Isolation Kit (Sigma Aldrich, USA). NaF exposed cells were lyzed using microRNA lysis buffer supplemented with RNA binding solution. miRNAs were isolated as per the instructions given in the manufactures protocol. 500 ng of miRNA was converted to cDNA using NCode VILO miRNA cDNA Synthesis Kit (Invitrogen, USA). Relative quantification of miRNA was performed on Applied Biosystems 7300 real time PCR system using EXPRESS SYBR GreenER qPCR SuperMix containing universal reverse primers (Invitrogen, USA) and forward customized primers of miR-124/miR-155 (Table 1). RUN48 was used as internal controls as described earlier [14]. Fold change was calculated using ddCT method (2^∧^-ddCt).

For validation of genes, total RNA was isolated using Trizol reagent. 2 *μ*g of total RNA was converted to cDNA using High Capacity Reverse Transcription kit (Applied Biosystems, USA). qPCR primers for RUNX2, RANKL, BGLAP, and OPG were used as described earlier (Table 1) [15–17]. RNA polymerase II (RPII) and hypoxanthine phosphoribosyl transferase (HPRT) were used as internal controls as described earlier [18]. Relative quantification was performed on Applied Biosystems 7300 real time PCR system using EXPRESS SYBR GreenER qPCR SuperMix. All the primers were validated before relative quantification. Fold change was calculated using ddCT method (2^∧^-ddCt).

### 2.7. C/D Box Analysis of snoRNA

Differentially expressed snoRNAs obtained by microarray analysis were further analyzed for the expression levels of C/D boxes using an in silico approach. Probes mapped on the array showed high length variability encompassed in the range from 48 to 237 bp. This great variability suggested partitioning the C/D box data set on the basis of their length. Box plot (Supplementary data; S3A) shows a statistical analysis of length outliers of C/D box snoRNAs. The length outliers in snoRNA were determined by interquartile range method, described below:

(1) $$\begin{array}{c} \text{Lower}\;\;\text{Limit}={\hat{q}}_{0.25}-1.5\;\text{IQR},\\ \text{Upper}\;\;\text{Limit}={\hat{q}}_{0.75}+1.5\;\text{IQR}. \end{array}$$

Lower and upper limits set for outlier detection were LL = 45.87 and UL = 112.87, respectively (please refer to supplementary data; S3 for the details).

## 3. Results

**Figure 1:**
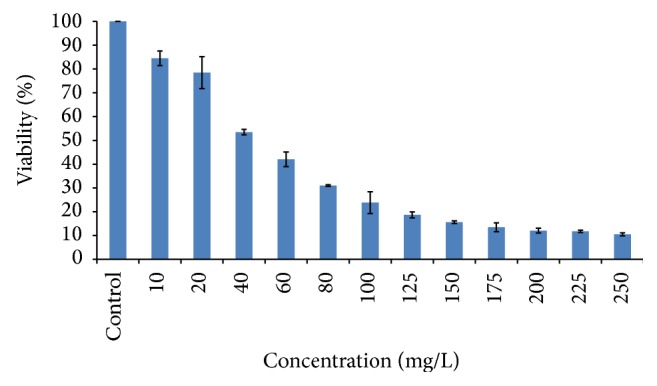
LC_50_ (24 h) of NaF against HOS cells. Graph represents the cytotoxicity caused by sodium fluoride after 24 h of exposure.

Cell viability was found to be decreased with increasing concentrations of NaF. LC_50_ (24 h) concentration of NaF against HOS cells was found to be 40 mg/L (Figure 1). Based on these results, sublethal concentrations (8 mg/L (1/5th) and 20 mg/L (1/2nd) of 24 h LC_50_) of NaF were chosen for further experiments.

**Figure 2:**
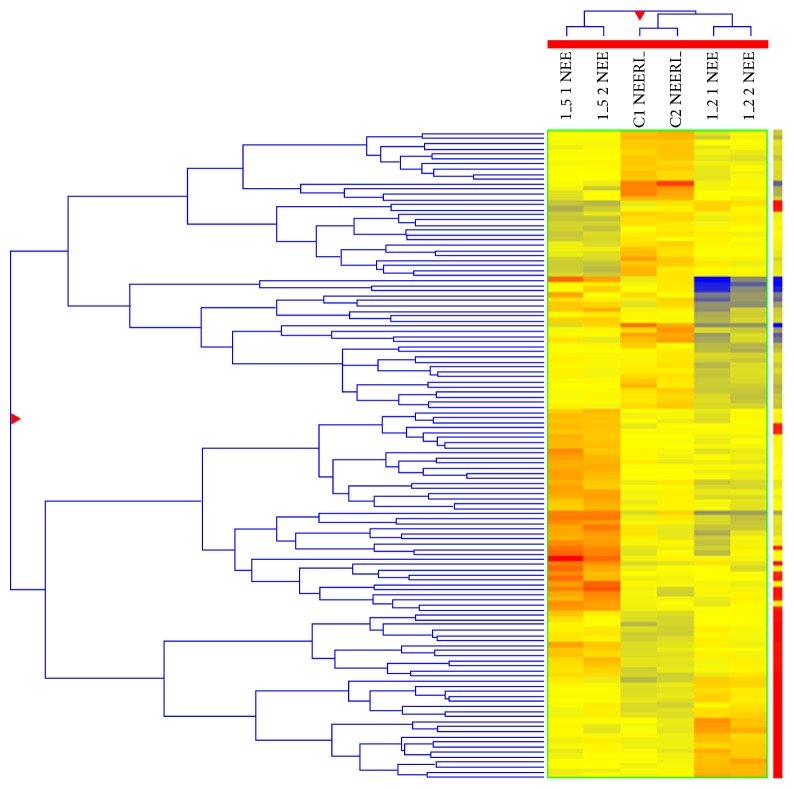
A Screen shot of Hcl clustering expression image of miRNAs. Red color shows overexpressed miRNAs (>0) and blue color shows underexpressed miRNAs (<0). The hcl heat map image has been generated on the basis of log2 normalized intensity value. Here, C1 and C2 represent controls. 1_5 and 1_2 represent 8 mg/L and 20 mg/L NaF concentrations, respectively.

The microarray experiment (Figure 2) was carried out using two different test concentrations, that is, 8 mg/L and 20 mg/L of NaF. Out of 5,639 probes, each test concentration displayed 128 differentially expressed miRNAs (*P* < 0.05). Supplementary Table S2 displays representative miRNA signatures which were differentially expressed in both the test concentrations (*P* < 0.05). Further screening with one and half fold change cut (FC ≥ 1.5) showed 82 and 62 differentially expressed miRNAs in 8 mg/L and 20 mg/L test concentrations of NaF, respectively. In it, 56 and 21 miRNAs were found to be upregulated in 8 mg/L and 20 mg/L of test concentrations, respectively.

**Figure 3:**
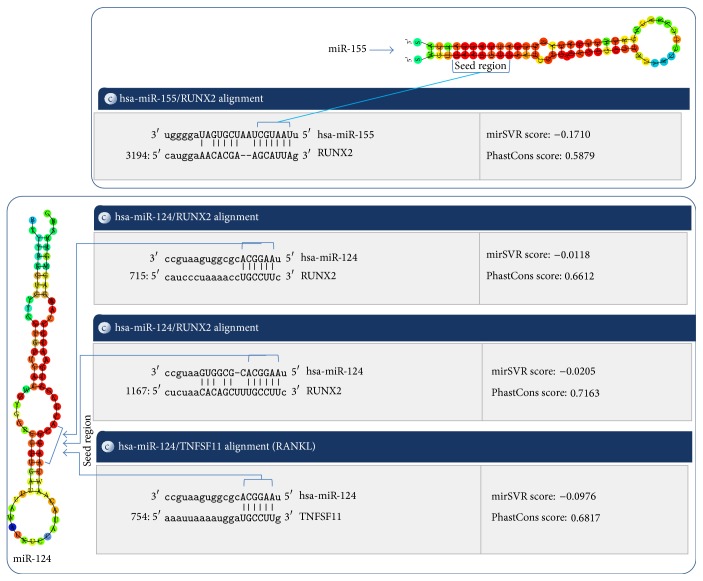
In silico hybridization analysis of mIR-124 and mIR-155 with the RUNX2 and RANKL.

**Figure 4:**
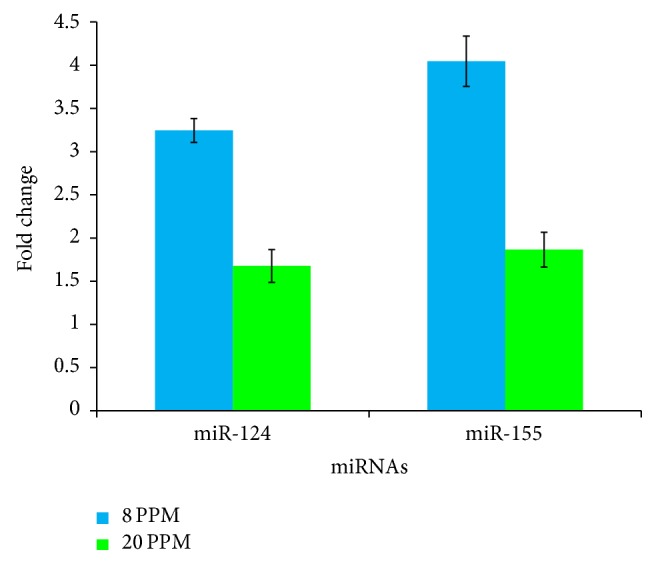
Validation of miRNAs by qPCR. The expression levels of miRNAs in NaF treated samples were normalized with the control.

mirTarBase and miRANDA software were used to understand the possible involvement of miRNAs in the regulation of osteoclastogenic signaling pathway. In silico hybridization revealed that seed regions miR-124 and miR-155 can bind with the untranslated regions (UTRs) of the RUNX2 and RANKL (Figure 3). Out of all the possible alignments of miR-124/miR-155 with the RUNX2/RANKL, pairs depicting hybridization with the conserved domains, good mirSVR and PhastCons scores were selected using miRANDA software. Validation of miRNAs using qPCR demonstrated that sublethal concentrations of NaF can significantly (*P* < 0.05) upregulate miR-124 and miR-155 expressions in HOS cells (Figure 4). Compared to the higher dose level (20 mg/L) of NaF, expression levels of miRNAs were more elevated in low dose level (8 mg/L). At lower dose level, fold change in the expression levels of miR-124 and miR-155 was observed to be 3.24 (±0.13) and 4.03 (±0.3), respectively. At higher dose level, fold change in the expression levels of miR-124 and miR-155 was observed to be 1.6 (±0.19) and 1.86 (±0.24), respectively.

**Figure 5:**
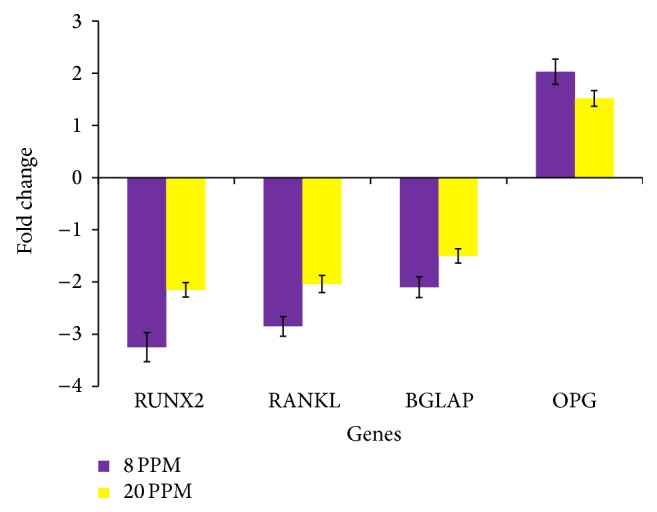
Validation of gene expression by qPCR. The expression levels of genes in NaF treated samples were normalized with the control.

Further validation through qPCR revealed that upregulation of miR-124 and miR-155 led to the downregulation of Runx2 (*P* < 0.05) in NaF treated HOS cells. At lower dose level, fold change in the expression levels of the RUNX2 was observed to be −3.25 (±0.27), whereas at higher dose level the fold change was observed to be −2.15 (±0.13) (Figure 5). Validation of other osteoblastic lineage markers (RANKL, BGLAP, and OPG) through qPCR suggested their possible correlation with the expression levels of miR-124 and miR-155. Expression levels of the RANKL and BGLAP were found to be significantly decreased (*P* < 0.05), whereas the expression level of the OPG was found to be significantly increased (*P* < 0.05) in NaF treated HOS cells. At lower dose level, fold change in the expression levels of the RANKL, BGLAP, and OPG was observed to be −2.85 (±0.18), −2.1 (±0.20), and 2 (±0.24), respectively. At higher dose level, fold change in the expression levels of the RANKL, BGLAP, and OPG was observed to be −2.04 (±0.16), −1.5 (±0.14), and 1.5 (±0.02), respectively (Figure 5).

Effects of sublethal concentrations of NaF on the expression profiles of 347 C/D box snoRNAs were analyzed using a quantile approach. C-box (UGAUGA) is typically located near the 5′ terminal of snoRNA, whereas the D-box (CUGA) is located in proximity of 3′ terminal. The identification of C/D box targets is quite different from miRNAs target screening. Identification of C/D box targets relies on the elucidation of putative methylation or pseudouridylation sites at chromosome level. Total snoRNA probes with different lengths (48–237 bp) were analyzed using an outlier analysis (Supplementary data; S3A). The upper length threshold of snoRNA probe was fixed to l ≥ 112.87. We have separately analyzed the statistical outliers in order to investigate their putative functions. Outlier analysis revealed 15 snoRNAs with different lengths ranging from (114–237 bps) (Supplementary data; S3B). In order to check the homogeneity of the longer C/D box snoRNAs, the average two-parameter kimura's distance was estimated for the putative outliers set (〈K2p〉 = 0.184) (Supplementary data; S3C). Further analysis revealed differential distribution of C-box and D-box (Supplementary data; S3D). Each snoRNA sequence, except U22 and U97, was found to contain one C-box, whereas numbers of D-box in the given set were found vary between 0 and 5. Majority of the length outliers (l > 112.87) of snoRNAs with two or more D-box sequences were found to be downregulated in NaF treated HOS cells compared to control. On the contrary, snoRNAs with less than two D-box sequences were found to be upregulated in NaF treated HOS cells compared to control (data not shown). Compared to CG dinucleotides, higher numbers of UG dinucleotides were found in the sequences of all outliers. Single channel microarray exploratory protocol analysis revealed up- and downregulation of C/D snoRNA dataset, including length outliers and without outliers (Supplementary data; S3E). 14qII-13 snoRNA was found to be downregulated at both the sublethal concentrations of NaF when analyzed with and without length outliers. 14qII-9, HBII-85-1, U42B, U53, U73b, and U84 snoRNAs were found to be upregulated at both the sublethal concentrations of NaF when analyzed with length outliers. In the dataset cleaned after outliers, the analyzed expression level of U58A snoRNA was found to be downregulated at both the sublethal concentrations of NaF, whereas at the same condition the expression levels of 14qII-9, HBII-336, HBII-52-7, U36B, U43, and U46 snoRNAs were found to be upregulated at both the sublethal concentrations of NaF. Rest of the snoRNAs (Supplementary data; S3E) were specifically expressed at either of the sublethal doses of NaF.

## 4. Discussion

At present, no successful therapeutics is available to treat fluorosis [2]. Available therapies against fluorosis mostly include supplementations (cofactor of enzymes, amino acids, and antioxidants) that can stimulate osteoblast/osteoclast mediated bone formation and remodeling processes, macrophage activation, and inhibition of reactive oxygen species (ROS) [2, 3]. However, the therapeutic effects incurred due to these therapies are not consistent. Reversals of the therapeutic effects are often seen once the medication is discontinued [2]. It is because these medications do not have control on the epigenetic modifications that are responsible for posttranscriptional regulation of genes involved in the osteoclastogenic pathway. Therefore, aim of the present study was to find out epigenetic modifications in miRNAs and C/D box snoRNAs that can regulate the key genes involved in the development of fluorosis.

Noncoding RNAs are known to regulate majority of the genes involved in the cellular pathways [9, 13, 14, 19]. Different xenobiotic compound may have different effects on the expression profiles of noncoding RNAs [19]. In the present study, sublethal concentrations of NaF were found to induce alterations in the expression profiles of noncoding RNAs (Figure 2). Functional targets of selected miRNAs (miR-124 and miR-155) were identified using mirTarBase and in silico hybridization analysis. Seed regions of the miR-124 and miR-155 displayed in silico hybridization with untranslated regions (UTRs) of the RUNX2. miR-124 displayed in silico hybridization with 3′ untranslated regions of RANKL (Figure 3). Further validation through quantitative RT-PCR showed that the expression profiles of the miR-124 and miR-155 were inversely proportional to the expression profiles of the RUNX2 and RANKL genes. Thus, it can be inferred that miR-124/miR-155 could be involved in the posttranscriptional regulation of the RUNX2 and RANKL in NaF exposed HOS cells.

Yin et al. (2010) studied role of miR-155 in regulation of BMP signaling pathway and demonstrated that miR-155 binds to 3′UTRs of target mRNAs and regulates the expression of key genes involved in BMP signaling pathway (RUNX2, SMAD1, SMAD5, HIVEP2, CEBPB, and MYO10) [10]. Further studies on knockout model demonstrated that miR-155 can directly inhibit BMP signalling pathway [10].

RUNX2 is an osteoblastic transcription factor that mediates osteoblast and osteoclast signaling pathway during bone formation and bone resorption [20]. Otto et al. reported that RUNX2 inhibition resulted in the death of new born null mouse due to the disfunctioning of bone formation and skeletal mineralization pathway [21]. Our results correspond with investigations on mice, which reported decrease in level of RUNX2 after NaF exposure [5, 22].

Significant decrease in expression levels of the RANKL was observed in NaF treated HOS cells (Figure 5). Similar results were reported in* Xenopus laevis* and B6 mice when exposed to sodium fluoride [5, 22, 23]. RANKL, a transmembrane protein, expresses on the osteoblast cells [6]. RANKL binds to its receptor (RANK) present on the progenitor osteoclast and stimulates osteoclastogenesis [6]. RANK-RANKL binding activate downstream target genes (TRAF6, NF-*κβ*, and C-fos) in RANK signaling pathway [4, 7, 8]. RANK-RANKL signaling facilitates phagocyte activation, osteoclast maturation, and bone resorption [4, 7, 8, 24]. Osteoclast at the end of remodeling step undergoes apoptosis via phagocytosis [4–8]. This results in the release of calcium in blood which assists the bone mineralization process. Available literature entails that RUNX2 acts as a transcription factor of RANKL [7], whereas our results based on qPCR and in silico hybridization analysis revealed that miR-124 can be involved in the direct regulation of RANKL (Figures 3, 4, and 5). Thus, it can be seen that miR-124/miR-155 can regulate the RANKL expression either through the direct binding of miR-124 with the RANKL untranslated regions or via RUNX2 mediated transcriptional inactivation.

Compared to the higher dose of NaF (20 mg/L), elevated levels of miR-124 and miR-155 expressions were seen at lower dose level of NaF (8 mg/L). It has been reported that fluoride exposure may have biphasic effect on osteoblast and osteoclast cells [2]. Fluoride exposure at lower concentration promotes bone development, whereas exposure to higher concentration of fluoride may lead to cellular stress [2]. Thus, the higher expression levels of miR-124 and miR-155 at lower concentrations of NaF (8 mg/L) obtained in our study could be attributed to the biphasic effects exerted by NaF. Compared to the higher dose level, elevated expression levels of miR-124 and miR-155 at lower dose of NaF resulted in the higher degree of differential expression of genes (RUNX2, RANKL, BGLAP, and OPG) involved in the osteoclastic differentiation.

**Figure 6:**
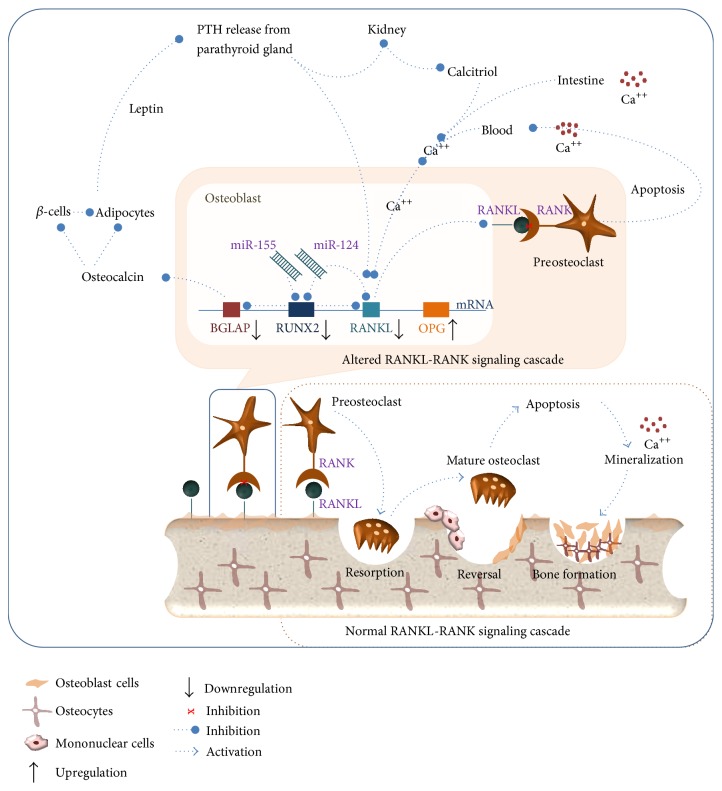
miRNA mediated alterations in bone formation and remodeling pathway.

Normal RANK-RANKL signaling provides proper structure and mechanical strength to bones [5, 6, 22]. However, study on various animal models suggests that increase in RANKL-RANK interaction resulted in osteoporosis, excessive loss of bone [24]. To regulate the interaction network between RANK-RANKL, osteoblast cells secret a soluble decoy of RANKL called OPG [23, 24]. Thus, excessive loss of bone can be prevented when OPG binds to RANKL and interferes with osteoblast and osteoclast interactions [23]. OPG and RANKL are synthesized by osteoblast cells. Decrease in RANKL or increase in OPG may result in osteopetrosis [23, 24]. In our study, OPG was found to be significantly upregulated, whereas the RANKL was found to be downregulated after NaF exposure to HOS cells (Figure 5). Thus, it can be seen that the sublethal concentrations of NaF can cause aberrant changes in the RANKL/RANK/OPG system. qPCR analysis revealed that the sublethal concentrations of NaF can downregulate the expression levels of BGLAP (osteocalcin) (Figure 5). Osteocalcin plays an important role in the process of bone mineralization, energy metabolism, and bone remodeling [25]. Carboxylated osteocalcin is involved in formation of calcium hydroxyapatite crystals which are deposited in the triple helical fibrils space of bone matrix [26]. Excess intake of fluoride may result in the downregulation of osteocalcin. It results in the conversion of calcium hydroxyapatite crystals to fluorapatite crystal [25, 26]. As a result, bones become stiff and loss its mechanical strength. Low concentration of osteocalcin can affect the expression of insulin and proliferation of *β*-cells [27]. This may limit leptin (secreted by adipocytes) mediated PTH (parathyroid hormone) release from parathyroid glands [27]. Low levels of PTH blocks 1-hydroxylase mediated upregulation of calcitriol (vitamin D) in the kidney [28]. As a result, process of absorption of dietary calcium in the small intestine and secretion of free calcium (via apoptosis of osteoclast) required for the RANKL mediated bone formation and remodeling is severely hampered (Figure 6) [27, 28].

Compared to CG dinucleotides, outlier analysis of C/D box snoRNAs revealed higher numbers of UG dinucleotides after NaF exposure to HOS cells. Spontaneous deamination of unmethylated cytosine residues of CG dinucleotides may result in the conversion of cytosine to uracil residues [13]. Deamination reaction is caused due to hydrolysis of cytosine [19]. Fluoride ion, being highly electronegative, can cause hydrolysis of water molecules inside the cellular environment. In this regard, the possibility of spontaneous deamination of unmethylated cytosine after the exposure of NaF cannot be denied.

Compared to the terminal C/D box, microarray analysis revealed more marked effect of fluoride exposure on the expression levels of internal C/D boxes of snoRNAs The C/D boxes snoRNAs are short ncRNAs that are mainly involved in RNA editing processes leaded by ADAR proteins. In particular the C/D box snoRNAs are prominently involved in methylation processes, at 2-O-ribose position of different cellular RNAs. Their name derives from the presence of two conserved nucleotide stretches: the C-box (UGAUGA) displaced near the 5 termini and the D-box (CUGA) located in proximity of 3 termini [29]. Many evidences support that one of the critical events, associated with environmental stress, is the alteration of methylation processes at DNA or RNA level. Recent studies have underlined the criticality of RNA methylation biological process such as cell stability [12].

Schroeder et al. reported that metal ions or binding proteins are responsible for stabilizing the minor grooves of the canonical and noncanonical stems of C-box and D-box motifs [30]. In this regard, longer C/D box snoRNAs may contain additional nucleotide motifs that can interact with the metals and other binding proteins and influence the functional role of these molecules. Deschamps-Francoeur et al. reported that snoRNA displaying long ends (snoRNA_L_) were overexpressed in ovarian and breast cancer cell lines, whereas snoRNA displaying short ends (snoRNA_SH_) were abundant in normal cell lines [31]. Furthermore, snoRNA_L_ were mostly found to display canonical snoRNA features compared to noncanonical features found in snoRNA_SH_. C/D box snoRNA may have an additional internal D-box in the middle of the molecule. Addition of internal D-box motifs can affect (1) hairpin structure formation of 5′ and 3′ motifs of C-box and D-box elements; (2) binding with contaminants; (3) expression of other noncoding RNAs. Thus, it can be stated that the conformational differences due to presence of varying numbers of C-box and D-box sequences may alter the functional motifs of C/D box snoRNAs.

Length outlier analysis showed high numbers of D-box containing snoRNAs in NaF treated HOS cells when compared to the numbers of C-box containing sequences (Supplementary data; S3D). Downregulation of C/D box length outliers with more numbers (≥2) of D-box sequences in NaF treated HOS cells suggest the possible interactions of NaF with snoRNAs due to conformational alterations exerted by additional D-box sequences. Microarray analysis revealed that sublethal concentrations of NaF can induce mark differences in the expression profiles of snoRNAs in HOS cells (Supplementary data; S3E). Structure of differentially expressed snoRNAs, their function, and reports on their possible interactions with osteoclastic pathway is given in Supplementary data S3F. Till date no reports are available on the possible interaction of snoRNAs in the osteoclastic pathway.

D-box has a vital role in the methylation of ribosomal RNA (rRNA) [32]. D-box recognizes the nucleotide of rRNAs to be methylated (usually 5th nucleotide upstream from the D-box binding site) [32, 33]. These results suggest that NaF can affect snoRNA mediated methylation of 28 s ribosomal RNA. This may result in the activation of aberrant transcriptional cascades involved in osteoclastogenic pathway. The present study was carried out only on a limited set of C/D box RNAs. This preliminary in silico analysis could be extended to whole set of currently identified C/D box RNAs for the further validation of the results obtained in this study.

## 5. Conclusion

Effect of fluoride on the epigenetic modifications is not yet reported. Herein, we report the direct/indirect possible interactions of mIR-124 and mIR-155 with the RUNX2 and RANKL in NaF treated HOS cells. Obtained results suggest that mIR-124 and mIR-155 can be considered the new players involved in the development of fluorosis. In addition, our results imply the possible role of C/D box snoRNAs in the development of fluorosis. However, these results need to be validated with the other in vitro and in vivo models. Future studies should be designed on the possible role of miRNAs (mIR-124 and mIR-155) and snoRNAs in the development of fluorosis and their implications as the possible therapeutic drug targets against fluorosis; in particular it is important to plan experiment to investigate the interactions between fluoride and proteins involved in the methylation cascade or in the miRNA dicer dependent and dicer independent biogenesis pathways.

## Supplementary Material

Supplementary data containing the detailed information on box whisker plot analysis, list of representative differentially expressed miRNAs and C/D box analysis of snoRNA.

## Figures and Tables

**Table 1:** qPCR primers.

Sr. number	Gene name	Primer	Sequence (5′-3′)
miRNAs			
1	miRNA-155	Forward primer	TGTTAATGCTAATATGTAGGAG
2	miRNA-124	Forward primer	GGCATTCACCGCGTGCCTTA
3	RUN48	Forward primer	AGTGATGATGACCCCAGGTAACTC
		Reverse primer	CTGCGGTGATGGCATCAG

Genes			
4	RUNX2	Forward primer	GGCAGGCACAGTCTTCCC
		Reverse primer	GGCCCAGTTCTGAAGCACC
5	RANKL	Forward primer	TCGTTGGATCACAGCACATCA
		Reverse primer	TATGGGAACCAGATGGGATGTC
6	BGLAP	Forward primer	ATGAGAGCCCTCACACTCCTC
		Reverse primer	GCCGTAGAAGCGCCGATAGGC
7	OPG	Forward primer	CTGGAACCCCAGAGCGAAAT
		Reverse primer	GCCTCCTCACACAGGGTAAC
5	RPII (internal control)	Forward primer	GCACCACGTCCAATGACAT
		Reverse primer	GTGCGGCTGGTTCCATAA
6	HPRT (internal control)	Forward primer	ACGAAGTGTTGGATATAAGC
		Reverse primer	ATAATTTTACTGGCGATGTC

## Abbreviations

NaF: Sodium fluoride
RUNX2: Runt-related transcription factor 2
RANKL: Receptor activator of nuclear factor kappa-B ligand
BGLAP: Bone gamma-carboxyglutamic acid-containing protein
OPG: Osteoprotegerin
RANK: Receptor activator of nuclear factor *κ*-B
miRNA: MicroRNAs
snoRNAs: Small nucleolar RNAs
PTH: Parathyroid hormone.

## References

[1] Deoki N., Mathiyazhagan T., Ambika P., Ravi T. (2007). An overview of fluoride and fluorosis. *Newsletter National Institute of Health and Family Welfare*.

[2] Everett E. T. (2011). Fluoride's effects on the formation of teeth and bones, and the influence of genetics. *Journal of Dental Research*.

[3] Erdal S., Buchanan S. N. (2005). A quantitative look at fluorosis, fluoride exposure, and intake in children using a health risk assessment approach. *Environmental Health Perspectives*.

[4] Anna T. (2011). Bone development: overview of bone cells and signaling. *Current Osteoporosis Reports*.

[5] Carvalho J. G., Leite A. L., Yan D., Everett E. T., Whitford G. M., Buzalaf M. A. R. (2009). Influence of genetic background on fluoride metabolism in mice. *Journal of Dental Research*.

[6] O'Brien C. A. (2010). Control of RANKL gene expression. *Bone*.

[7] Joana  C. L., Helena C. J., Eurico F. (2007). Osteoblasts and bone formation. *Acta Reumatológica Portuguesa*.

[8] Avnet S., Pallotta R., Perut F. (2011). Osteoblasts from a mandibuloacral dysplasia patient induce human blood precursors to differentiate into active osteoclasts. *Biochimica et Biophysica Acta—Molecular Basis of Disease*.

[9] Jian H., Lan Z., Lianping X., Di C. (2010). MicroRNA-204 regulates Runx2 protein expression and mesenchymal progenitor cell differentiation. *Stem Cells*.

[10] Yin Q., Wang X., Fewell C. (2010). MicroRNA miR-155 inhibits bone morphogenetic protein (BMP) signaling and BMP-mediated epstein-Barr Virus Reactivation. *Journal of Virology*.

[11] Li Z., Hassan M. Q., Volinia S. (2008). A microRNA signature for a BMP2-induced osteoblast lineage commitment program. *Proceedings of the National Academy of Sciences of the United States of America*.

[12] Stepanov G. A., Semenov D. V., Savelyeva A. V. (2013). Artificial box C/D RNAs affect pre-mRNA maturation in human cells. *BioMed Research International*.

[13] Scott M. S., Ono M., Yamada K., Endo A., Barton G. J., Lamond A. I. (2012). Human box C/D snoRNA processing conservation across multiple cell types. *Nucleic Acids Research*.

[14] Nurul S. A., Lee L. H., Shiran M. S., Sabariah A. R., Avatarsingh M. S., Cheah Y. K. (2012). miR-181a regulates multiple pathways in hypopharyngeal squamous cell carcinoma. *African Journal of Biotechnology*.

[15] Nimura A., Muneta T., Otabe K. (2010). Analysis of human synovial and bone marrow mesenchymal stem cells in relation to heat-inactivation of autologous and fetal bovine serums. *BMC Musculoskeletal Disorders*.

[16] Luo X.-H., Guo L.-J., Xie H. (2006). Adiponectin stimulates RANKL and inhibits OPG expression in human osteoblasts through the MAPK signaling pathway. *Journal of Bone and Mineral Research*.

[17] Sun X., Wei L., Chen Q., Terek R. M. (2009). HDAC4 represses vascular endothelial growth factor expression in chondrosarcoma by modulating RUNX2 activity. *The Journal of Biological Chemistry*.

[18] Sain S., Naoghare P. K., Devi S. S. (2014). Beta caryophyllene and caryophyllene oxide, isolated from Aegle marmelos, as the potent anti-inflammatory agents against lymphoma and neuroblastoma cells. *Anti-Inflammatory & Anti-Allergy Agents in Medicinal Chemistry*.

[19] Valleron W., Laprevotte E., Gautier E. F. (2012). Specific small nucleolar RNA expression profiles in acute leukemia. *Leukemia*.

[20] Liu J. C., Lengner C. J., Gaur T. (2011). Runx2 protein expression utilizes the Runx2 P1 promoter to establish osteoprogenitor cell number for normal bone formation. *Journal of Biological Chemistry*.

[21] Otto F., Thornell A. P., Crompton T. (1997). Cbfa1, a candidate gene for cleidocranial dysplasia syndrome, is essential for osteoblast differentiation and bone development. *Cell*.

[22] Yan D., Gurumurthy A., Wright M., Pfeiler T. W., Loboa E. G., Everett E. T. (2007). Genetic background influences fluoride's effects on osteoclastogenesis. *Bone*.

[23] Nair M., Belak Z. R., Ovsenek N. (2011). Effects of fluoride on expression of bone-specific genes in developing *Xenopus laevis* larvae. *Biochemistry and Cell Biology*.

[24] Jones D. H., Kong Y. Y., Penninger J. M. (2002). Role of RANKL and RANK in bone loss and arthritis. *Annals of the Rheumatic Diseases*.

[25] Roberto P., Gloria A., Fernando C. (2004). Bone-specific transcription factor Runx2 interacts with the1*α*, 25-dihydroxyvitamin D3 eceptor to up-regulate rat osteocalcin gene expression in osteoblastic cells. *Molecular and Cellular Biology*.

[26] Doherty T. M., Fitzpatrick L. A., Inoue D. (2004). Molecular, endocrine, and genetic mechanisms of arterial calcification. *Endocrine Reviews*.

[27] Gilberto P., Claudio M., Ma-Li W., Julio L. (2012). Leptin therapy, insulin sensitivity, and glucose homeostasis. *Indian Journal of Endocrinology and Metabolism*.

[28] Patti A., Gennari L., Merlotti D., Dotta F., Nuti R. (2013). Endocrine actions of osteocalcin. *International Journal of Endocrinology*.

[29] Lin J., Lai S., Jia R. (2011). Structural basis for site-specific ribose methylation by box C/D RNA protein complexes. *Nature*.

[30] Schroeder K. T., Mcphee S. A., Ouellet J., Lilley D. M. J. (2010). A structural database for k-turn motifs in RNA. *RNA*.

[31] Deschamps-Francoeur G., Garneau D., Dupuis-Sandoval F. (2014). Identification of discrete classes of small nucleolar RNA featuring different ends and RNA binding protein dependency. *Nucleic Acids Research*.

[32] Lapinaite A., Simon B., Skjaerven L., Rakwalska-Bange M., Gabel F., Carlomagno T. (2013). The structure of the box C/D enzyme reveals regulation of RNA methylation. *Nature*.

[33] Brameier M., Herwig A., Reinhardt R., Walter L., Gruber J. (2011). Human box C/D snoRNAs with miRNA like functions: expanding the range of regulatory RNAs. *Nucleic Acids Research*.

