# Supplementary material for: Noncoding RNAs: Possible Players in the Development of Fluorosis
Source: Biomed Res Int. 2015 Aug 3;2015:274852. doi: 10.1155/2015/274852 (PMC4538412; doi:10.1155/2015/274852)
Supplement: Supplementary file 1 — Supplementary data containing the detailed information on box whisker plot analysis, list of representative differentially expressed miRNAs and C/D box analysis of snoRNA. [file 274852.f1.docx]

**S1.** Box whisker plot analysis

The total number of Homo sapiens miRNAs probesets detected for the experiment is 5,639. The Box whisker plot (Supplementary Figure 1) presents the normalized microarray expression data visualization summary. Further data is also distributed on conditions in the active interpretation with respect to the active entity list in the experiment. The box whisker plots are created between normalized intensity values and all probes. The box whisker shows the median in the middle of the box, the 25th quartile and the 75th quartile. After this all normalized data imported in Gene Spring 12.5 for further analysis.

**Figure 1.** Box whisker plot analysis. Data was normalized using RMA with median transform.


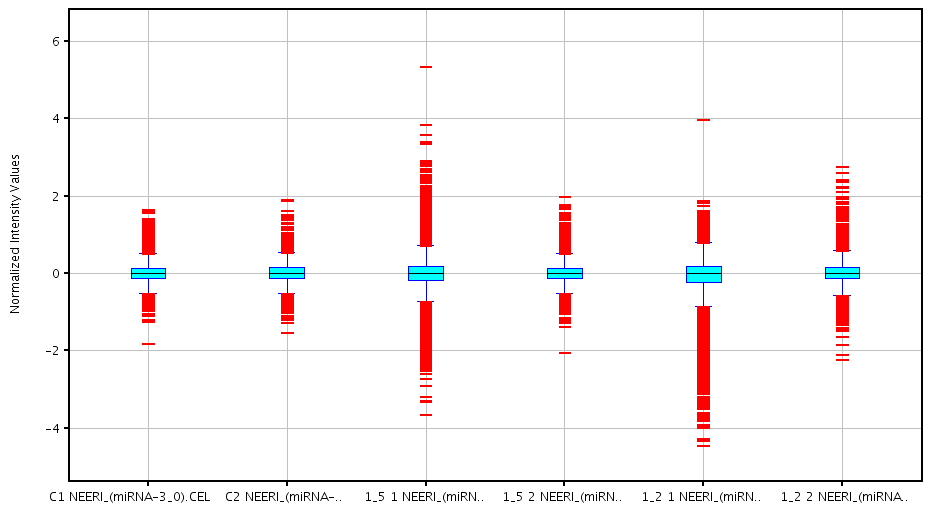


Control 1 Control 2 8mg/l NaF 8mg/l NaF 20mg/l NaF 20mg/l NaF

**S2.** List of representative differentially expressed miRNAs in both the test concentrations.

| Probe Set ID | Mirbase ID | Fold change (dose 8mg/L) | Fold Change (dose 20mg/L) |
| --- | --- | --- | --- |
| hp_hsa-let-7b_x_st | hsa-let-7b | 1.7126837 | -1.1148164 |
| hp_hsa-mir-1298_st | hsa-mir-1298 | 1.8202779 | -1.0515273 |
| hp_hsa-mir-16-2_st | hsa-mir-16-2 | 1.7897862 | -1.2196014 |
| hp_hsa-mir-2114_st | hsa-mir-2114 | -1.1506293 | -1.7440094 |
| hp_hsa-mir-3615_st | hsa-mir-3615 | -1.2958708 | -1.9804242 |
| hp_hsa-mir-4523_st | hsa-mir-4523 | -1.2387203 | -1.8235269 |
| hp_hsa-mir-548e_st | hsa-mir-548e | 2.7770593 | 1.0377077 |
| hp_hsa-mir-548f-1_st | hsa-mir-548f-1 | 2.4550493 | -1.4797523 |
| hp_hsa-mir-548h-2_x_st | hsa-mir-548h-2 | 2.3696961 | -1.110064 |
| hp_hsa-mir-609_st | hsa-mir-609 | 2.1324365 | 1.1032805 |
| hp_hsa-mir-760_st | hsa-mir-760 | -1.1619223 | -2.059323 |
| hsa-miR-1181_st | hsa-mir-1181 | -2.1564412 | -1.3747437 |
| hsa-miR-1183_st | hsa-mir-1183 | 1.8937967 | -1.5065389 |
| hsa-miR-1193_st | hsa-mir-1193 | 2.333104 | 1.1204691 |
| **hsa-miR-124_st** | **hsa-mir-124-1, 2, 3** | **2.0037158** | **1.0601381** |
| hsa-miR-1273d_st | hsa-mir-1273d | -1.9964552 | -1.2753638 |
| hsa-miR-1273f_st | hsa-mir-1273f | -2.7636507 | -2.152592 |
| **hsa-miR-155-star_st** | **hsa-mir-155** | **2.9648197** | **1.2978799** |
| hsa-miR-1909_st | hsa-mir-1909 | 1.064486 | -4.685758 |
| hsa-miR-1914-star_st | hsa-mir-1914 | -1.3737271 | -2.0141711 |
| hsa-miR-216b_st | hsa-mir-216b | 1.5669818 | 2.1242251 |
| hsa-miR-3132_st | hsa-mir-3132 | 2.319006 | -1.0967865 |
| hsa-miR-3173-3p_st | hsa-mir-3173 | 2.517634 | 1.2456092 |
| hsa-miR-3190_st | hsa-mir-3190 | 2.0196924 | -1.4448574 |
| hsa-miR-3622a-5p_st | hsa-mir-3622a | 1.1278787 | -3.8454146 |
| hsa-miR-3677-5p_st | hsa-mir-3677 | -1.9122614 | -1.7405812 |
| hsa-miR-371b-5p_st | hsa-mir-371b | 1.0735685 | -2.9778554 |
| hsa-miR-4261_st | hsa-mir-4261 | 1.8759298 | -1.2174667 |
| hsa-miR-4271_st | hsa-mir-4271 | 2.373002 | -2.2085476 |
| hsa-miR-4274_st | hsa-mir-4274 | -2.2768385 | -1.6578509 |
| hsa-miR-4321_st | hsa-mir-4321 | -1.833508 | -3.1079457 |
| hsa-miR-4428_st | hsa-mir-4428 | 2.3010745 | -4.145692 |
| hsa-miR-4468_st | hsa-mir-4468 | 2.0770998 | -1.3222356 |
| hsa-miR-4476_st | hsa-mir-4476 | 3.9630663 | -1.0778635 |
| hsa-miR-4633-5p_st | hsa-mir-4633 | -1.6192812 | -2.0469303 |
| hsa-miR-4673_st | -2.351784 | -1.6462884 | hsa-mir-4673 |
| hsa-miR-4716-3p_st | hsa-mir-4716 | 3.4312 | 1.2463163 |
| hsa-miR-4750_st | hsa-mir-4750 | 1.2383745 | -2.7507915 |
| hsa-miR-498_st | hsa-mir-498 | -1.722157 | -2.806436 |
| hsa-miR-570_st | hsa-mir-570 | 2.93191 | -1.1218857 |
| hsa-miR-665_st | hsa-mir-665 | -3.223474 | -3.045586 |
| hsa-miR-671-3p_st | hsa-mir-671 | -2.1530235 | -1.4284221 |

**S3.**C/D box analysis of snoRNA

1. **Box plot of 347 outlier C/D box snoRNAs.**


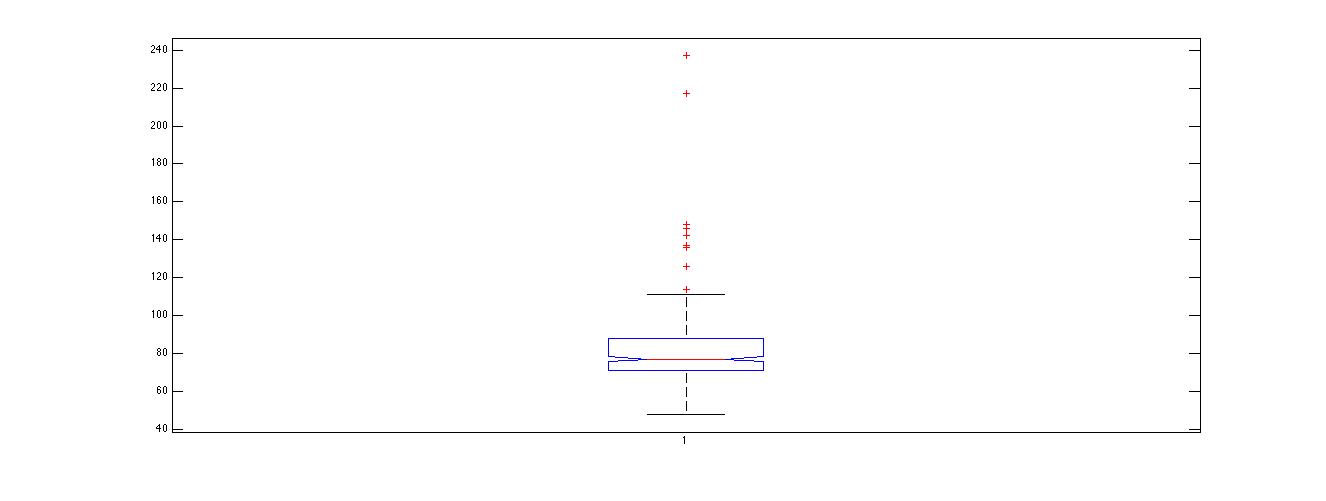


1. **Experimental evidence of outliers:**

Below-mentioned table displays the analysis of C/D box snoRNAs above the upper length limit ($\boldsymbol{l}\boldsymbol{\geq}\boldsymbol{112}\boldsymbol{.}\boldsymbol{87}\boldsymbol{)}$, length of snoRNAs and the experimental evidence on the respective snoRNAs.

| C/D Box Length outliers | Length (bp) | Experimental evidence |
| --- | --- | --- |
| HBI-43 | 237 | No |
| HBII-289 | 114 | PasinettiGM, Lap Ho et al. Am.J. of Neurodegener. Dis. 2012 88-98 |
| U15A | 148 | No |
| U15B | 146 | No |
| U22 | 126 | García JH, Osuna MD, Castrejon FM, Enriquez LG, Reyes PA, Hermosillo JJ. J Clin Lab Anal. 2004;18(1):19-26. |
| U3-2B | 217 | Padilla PI, Uhart M, Pacheco-Rodriguez G, Peculis BA, Moss J, Vaughan M.ProcNatlAcad Sci U S A. 2008 Mar 4;105(9):3357-61. |
| U3-2 | 217 |  |
| U3-3 | 217 |  |
| U3-4 | 217 |  |
| U3 | 217 |  |
| U8 | 136 | Scarsdale JN, Peculis BA, Wright HT. Structure. 2006 Feb;14(2):331-43. |
| U8 | 136 | No |
| U94 | 137 | Ifon ET, Pang AL, Johnson W, Cashman K, Zimmerman S, Muralidhar S, Chan WY, Casey J, RosenthalLJ.Cancer Cell Int. 2005 Jun, 22;5:19 |
| U97 | 142 | Theimer CA, Jády BE, Chim N, Richard P, Breece KE, Kiss T, Feigon J..Mol Cell. 2007 Sep 21;27(6):869-81. |
| mgU6-77 | 148 | Simpson PT, Reis-Filho JS, Lambros MB, Jones C, Steele D, Mackay A, Iravani M, Fenwick K, Dexter T, Jones A, Reid L, Da Silva L, Shin SJ, Hardisson D, Ashworth . J Pathol. 2008 Jul;215(3):231-44. |

1. **Cladogram of the outlier set**

**
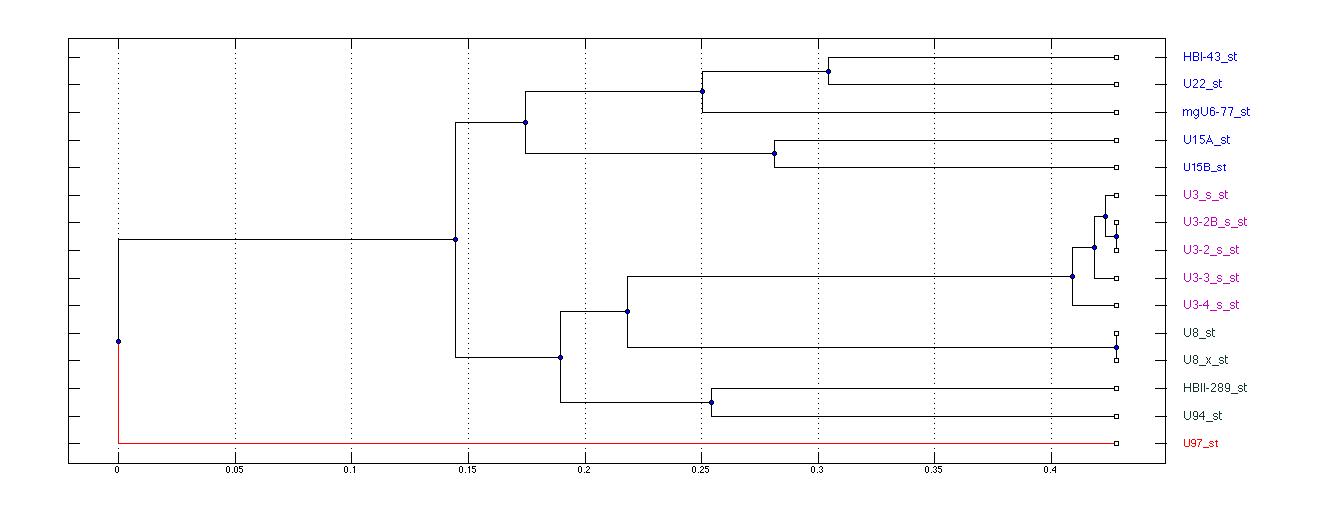
**The above cladogram point out the presence of two major groups of C/D box outliers and also the existence of one singleton elements (U97_st).

1. **Frequencies of C-box and D-box in length outliers**

| C/D box ncRNA  (length outliers)  $l>112.87$ | Number of D-box in the sequence (CUGA) | Number of C-box in the sequence (UGAUGA) | Number of  CG dinucleotide in the sequence | Number of UG  dinucleotide in the sequence |
| --- | --- | --- | --- | --- |
| HBI-43  HBII-289  U15A  U15B  U22  U3-2B  U3-2  U3-3  U3-4  U3  U8  U94  U97  mgU6-77 | 5  3  2  1  1  3  3  3  2  3  2  4  0  2 | 1  1  1  1  0  1  1  1  1  1  1  1  0  0 | 4  3  3  5  0  12  12  12  12  12  5  2  6  2 | 30  10  18  18  11  17  17  17  17  17  14  21  11  18 |

1. **Up and down regulation of differentially expressed C/D snoRNA probes in presence and absence of outliers.**

| **NaF 20mg/L** | **NaF 8mg/L** |
| --- | --- |
| **Down regulated C/D snoRNAs probes after NaF exposure (analysis of whole dataset including length outliers)** | |
| 14qII-11; **14qII-13**; 14qII-20; 14qII-2; HBII-108 (SNORD19); HBII-52-23 (SNORD115-23); HBII-52-39 (SNORD115-39); HBII-13 (SNORD64**)** | 14qI-1; **14qII-13**; 14qII-14; 14qII-18; 14qII-29; 14qII-3; HBII-85-10; mgU6-77 (SNORD10) |
| **Up regulated C/D snoRNAs probes after NaF exposure (analysis of whole dataset including length outliers)** | |
| 14qII-12; 14qII-25; **14qII-9**; HBII-13 (SNORD64); HBII-240 (SNORD72); HBII-289 (SNORD89); HBII-419 (SNNORD98); HBII-52-13 (SNORD115-13); HBII-85-15 (SNORD116-15); HBII-85-17 (SNORD116-17); HBII-85-19 (SNORD116-19); **HBII-85-1** (SNORD116-1); HBII-85-21 (SNORD116-21); HBII-85-7 (SNORD116-7); HBII-99B_s; U102; U18A; U22; U31; U35B; **U42B**; U47; **U53**; U61; **U73b**; U83A; **U84**; mgh18S-121; snR38B | **14qII-9**; HBII-234; **HBII-85-1**; HBII-85-5; U14A; **U42B**; **U53**; **U73b**; **U84**; U96b; mgh18S-121; HBII-296B; U86; 14qII-5 |
| **Down regulated C/D snoRNAs probes after NaF exposure (analysis of whole dataset without length outliers)** | |
| 14qII-11; **14qII-13**; 14qII-20; 14qII-2; HBII-437; U49A_s; U53; **U58A**; U79; HBII-239; SNORD121A; U26 | 14qI-1; **14qII-13**; 14qII-14; 14qII-18; 14qII-29; HBII-52-2; U38B; U45C; **U58A**; U86; mgU6-53; snR38A; U59B |
| **Up regulated C/D snoRNAsprobes after NaF exposure (analysis of whole dataset without length outliers)** | |
| mgU6-53; 14qII-25; **14qII-9**; HBII-436; HBII-85-7; U101; 14qII-12; HBII-296A; **HBII-336**; HBII-420; HBII-52-1; HBII-52-29; HBII-52-35; HBII-52-37; HBII-52-3; **HBII-52-7**; HBII-52-8; HBII-85-26_s; U106; U21; U27; **U36B**; **U43**; U44; **U46** | 14qII-5; **14qII-9**; HBII-336; HBII-85-7; 14qII-9; HBII-202; **HBII-336**; HBII-52-34; HBII-52-37; **HBII-52-7**; HBII-85-11; HBII-85-20; HBII-85-28; U102; U13; **U36B; U43**;  **U46** |

1. **Structure of differentially expressed snoRNAs, function and possible interactions with osteoclastic pathway. snoRNA structure were taken from Rfam data base.**

| Structure of snoRNA | Function | Available reports on the interaction of snoRNAs with osteoclastic pathway |
| --- | --- | --- |
| **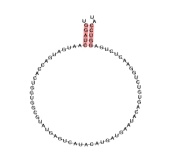14qII-13** | 2'-O-ribose methylation and Pseudouridylation of rRNA | No |
| **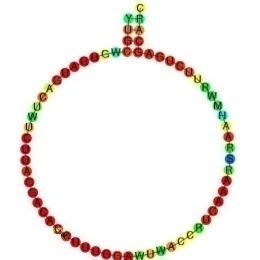U58A** | 2'-O-ribose methylation and Pseudouridylation of rRNA | No |
| **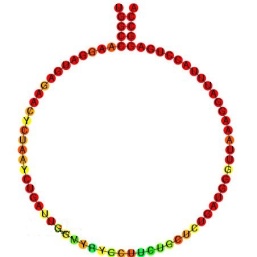HBII-336** | 2'-O-ribose methylation and Pseudouridylation of rRNA | No |
| 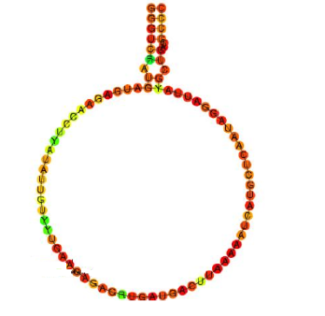**HBII-52-7** | 1. Autism 2. HBII-52 expression changes alternative splicing of DPM2, TAF1, RALGPS1, PBRM1 and CRHR1 . 3. Affect serotonin 2C receptor (5-HT2CR) correlated with altered Ca2+ responses elicited by a 5-HT2CR 4. 2'-O-ribose methylation and Pseudouridylation of rRNA | No |
| **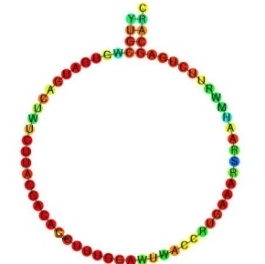U36B** | 2'-O-ribose methylation and Pseudouridylation of rRNA | No |
| **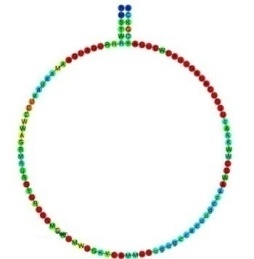U46** | 2'-O-ribose methylation and Pseudouridylation of rRNA | No |
|  **HBII-85-1** | 1. Growth retardation 2. Prader-willi syndrome 3. Obesity, impaired growth and hypogonadism | No |
| **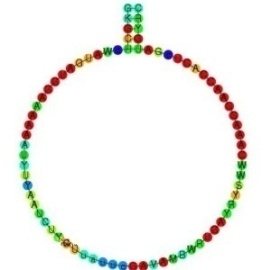U42B** | 2'-O-ribose methylation and Pseudouridylation of rRNA | No |
| **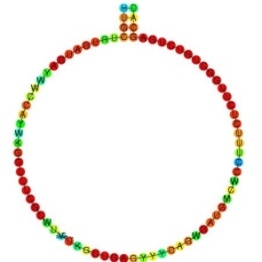U53** | 2'-O-ribose methylation and Pseudouridylation of rRNA | No |
| **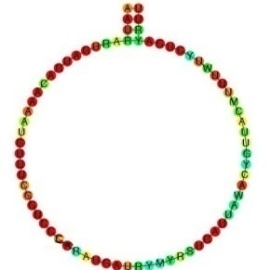U73b** | 2'-O-ribose methylation and Pseudouridylation of rRNA | No |
| **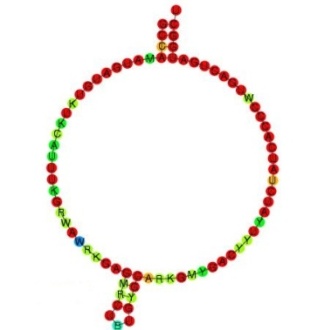U84** | 2'-O-ribose methylation and Pseudouridylation of rRNA | No |
